# No increase in the CTG repeat size during transmission from parent with expanded allele: false suspicion of contraction phenomenon

**DOI:** 10.1515/almed-2022-0079

**Published:** 2023-03-06

**Authors:** Nuria Goñi Ros, Paula Sienes Bailo, Ricardo González Tarancón, Loreto Martorell Sampol, Silvia Izquierdo Álvarez

**Affiliations:** Servicio de Genética y Bioquímica Clinica, Hospital Universitario Miguel Servet, Zaragoza, Spain; Servicio de Medicina Genética y Molecular, Hospital Sant Joan de Déu, Barcelona, Spain

**Keywords:** *DMPK*, myotonic dystrophy type 1, TP-PCR limitations, trinucleotide repeats

## Abstract

**Objectives:**

Myotonic dystrophy type 1 (DM1), also known as Steinert’s disease, is a chronic, progressive and disabling multisystemic disorder with a broad spectrum of severity that arises from an autosomal-dominant expansion of the Cytosine-Thymine-Guanine (CTG) triplet repeat in the 3′ untranslated region of the *DMPK* gene (19q13.3).

**Case presentation:**

In this study, we report the case of a family with several intergenerational expansions of the CTG repeat, with an additional case of a false suspicion of contraction phenomenon due to TP-PCR limitations.

**Conclusions:**

The meiotic instability of the (CTG)_n_ repeats leads to genetic anticipation where increased size of DM1 mutation and a more severe phenotype have been reported in affected individuals across generations. Even if extremely rare, a decrease in the CTG repeat size during transmission from parents to child can also occur, most frequently during paternal transmissions.

## Introduction

Myotonic dystrophy type 1 (DM1; OMIM#160900) is a chronic, progressive and disabling multisystemic disorder with a broad spectrum of severity that arises from an autosomal-dominant expansion of the cytosine-thymine-guanine (CTG) triplet repeat in the 3′ untranslated region of the serine-threonine kinase *DMPK* gene on chromosome 19q13.3. The resulting RNA becomes toxic due to the expanded CUG repeats forming ribonuclear foci, comprised of hairpin structures that bind and sequester RNA-binding proteins [1].

DM1, also known as Steinert’s disease, with a worldwide prevalence of approximately 1 in 8,000, is the most common type of adult-onset muscular dystrophy. Patients with DM1 typically experience skeletal weakness and wasting, muscle hyperexcitability (myotonia), cataracts, cognitive impairment, gastrointestinal problems, cardiovascular complications and insulin resistance, amongst other symptoms [2]. As the phenotypic spectrum is wide, and symptoms are variable, the identification and validation of suitable outcome measures for clinical research is quite challenging [3]. While in healthy subjects, CTG repeats range from 5 to 37, in individuals suffering from Steinert’s disease, this CTG triplet is repeated from 50 to more than 3,000. The repeat lengths of 38–50 CTGs are considered premutation alleles, which show increased instability towards larger pathologically expanded repetitions. The meiotic instability of the (CTG)_n_ repeats leads to genetic anticipation where increased size of DM1 mutation and a more severe phenotype have been reported in affected individuals across generations [4]. Generally, the higher the number of repeats, the greater the severity of the disease. Even though there is no exact parallel between these two factors, genotype–phenotype correlation is greater over 400 CTG repeats. Various diagnostic classifications have been proposed including congenital, infantile/childhood, juvenile, adult-onset and later adult/asymptomatic, each of them based on age of onset and severity of the symptoms [5, 6]. Even if rare, a decrease in the CTG repeat size during transmission from parents to child can also occur, most frequently during paternal transmissions [7]. TP-PCR is an effective alternative to the limitations of other current diagnostic methods such as Southern blot and conventional PCR, as these require large sample volumes, higher DNA concentrations, greater handling and consequently longer response times. In addition, conventional PCR do not distinguish between homozygotes/heterozygotes, and neither of them clearly detects mosaicisms with alleles of different sizes. TP-PCR allows an accurate quantification of alleles in the range of 5–150 CTGs repeats. Conventional agarose gel PCR can quantify up to about 100 CTGs repeats, but the accuracy in the size of the number of repeats is lower than TP-PCR. While with Southern blotting, the error is usually greater for alleles in that range; for expansion sizes of >150 CTGs, it is the best option, as it allows an accurate quantification of the number of repeats. Therefore, the main limitation of TP-PCR is that it does not allow quantification of the number of repeats of the expanded alleles (in the case of Steinert’s disease for alleles with a size greater than 150 repeats).

In this study, we report the case of a family with several intergenerational expansions of the CTG repeat, with the additional finding of a child with a false suspicion of contraction of the expanded DM1 allele due to TP-PCR limitations.

## Case presentation

**Figure 1:**
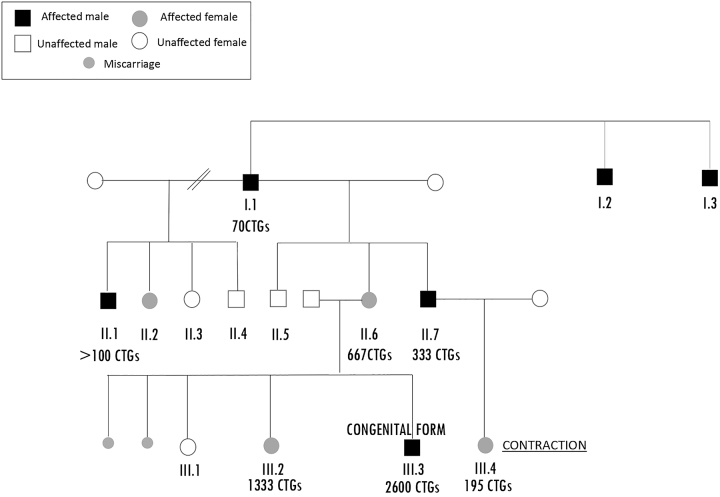
Familial pedigree with *DMPK* CTG genotype.

The index case (Figure 1. II.6) was a 33-year-old woman, 23 weeks pregnant, with myopathic facies, myotonic phenomenon in hands, asthenia/tiredness and inverted V-shaped upper lip. As family history of interest, her father and grandparent suffered from eye cataracts and she had a brother with similar symptoms. Due to the absence of relationship with the paternal branch of the family, other possible data of interest were unknown. She had suffered two miscarriages at the time of the consultation, and one of her daughters showed developmental delay and hypotonia. With a suspicion of Steinert’s disease, neurologists requested a genetic study to confirm or rule out the presence of variants in *DMPK* gene that would support the diagnosis of DM1. In our laboratory, techniques of PCR (Adellgene Myotonic Dystrophy Screening kit, Diagnóstica Longwood) and Triplet Primed Repeat PCR, TP-PCR (Adellgene^®^ Myotonic Dystrophy Confirmatory kit, Diagnóstica Longwood) are used for this purpose. Fragment analysis was carried out by the sequencer ABI 3130xl and the GeneMapper 4.0 software. Allele size detection limit by TP-PCR was 150 CTG, and the accuracy of PCR sizing was  ± 1 repeat for the normal allele size range. In this case, the TP-PCR report revealed a result compatible with the diagnosis of Steinert’s disease, as an allele of about 5 ± 1 CTG repeats and a second expanded allele >150 CTGs (about 667 CTGs, confirmed by Southern-blot) were found in the patient’s sample.

At this point, it was considered a genetic study of the daughters of our index case, to analyse the presence of alleles associated with DM1. In one of them (Figure 1. III.1), two alleles were found within the normal range (6 ± 1 CTGs, and 13 ± 1 CTGs), ruling out the disease. The other (Figure 1. III.2) was a 5-year-old girl with psychomotor retardation and hypotonia, whose study result revealed one allele with >150 CTGs, confirmed by Southern blot, quantifying 1,333 CTG repeats. Since the woman was pregnant at the time of diagnosis, a prenatal study was also performed, and it allowed us to detect a congenital form of the disease (Figure 1. III.3) with one allele of 2,600 CTG repeats. Despite this, the couple decided to continue with pregnancy. Their son was born with hypotonia, respiratory acidosis, breathing difficulties and hyperbilirubinemia. With 15 days of life, he required parenteral nutrition and combined tracheostomy and gastrostomy. He finally died at 4 months of age due to bradycardia and hypoperfusion.

**Figure 2:**
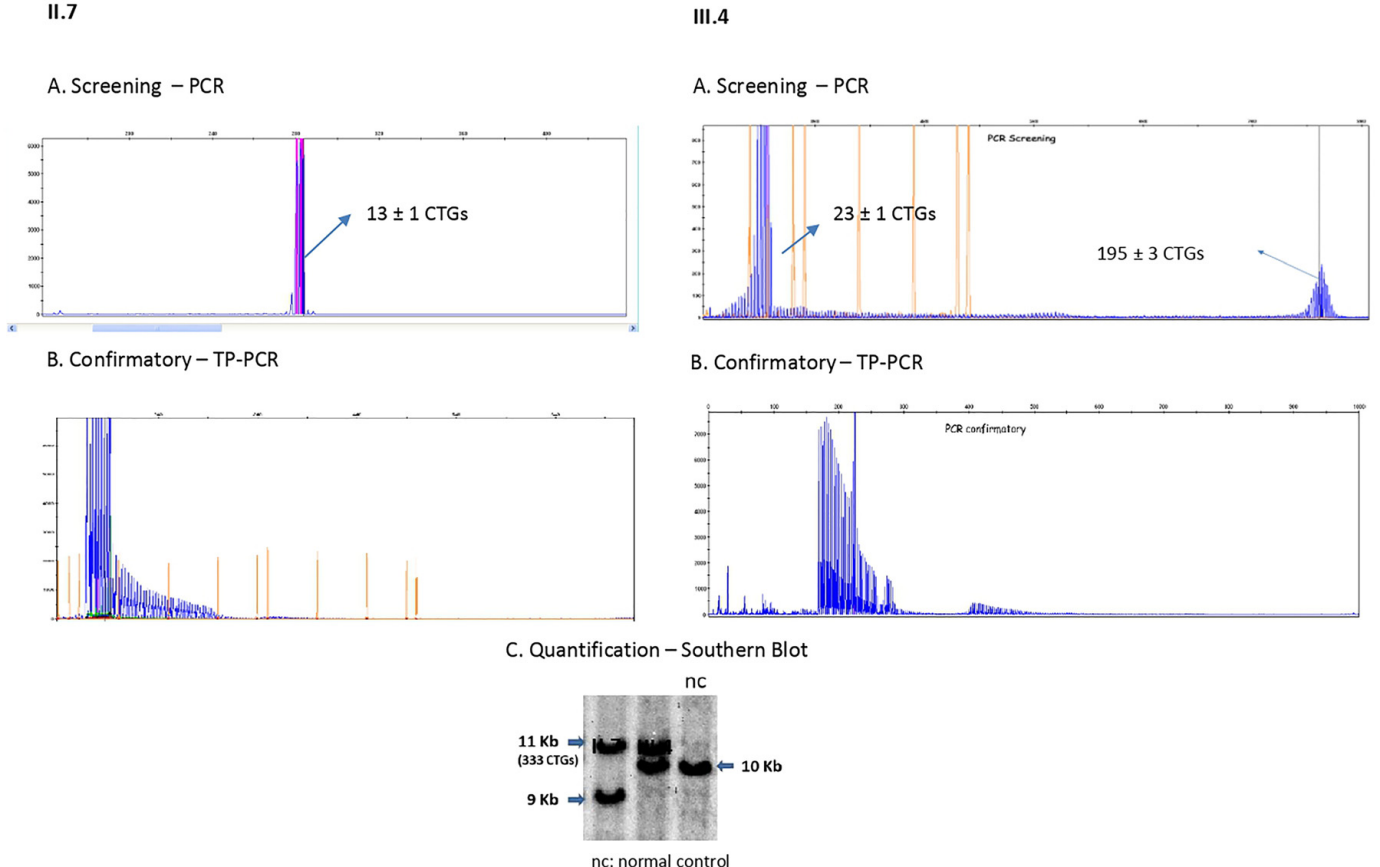
Results of genetic testing in II.7 and III.4 individuals (screening-PCR, confirmatory-TP-PCR and quantification-Southern blot).

Following this case of a congenital form of DM1, a genetic study was carried out on the child’s uncle (brother of our index case) (Figure 1. II.7). He was a 29-year-old male with myotonic phenomenon in face and frontal baldness. As results, we found one allele within the normal range, and another expanded (333 CTGs). The patient and his wife were expecting a baby, so we continued with the family study, with the aim of finding out if the baby was also a carrier of the CTG trinucleotide expansion in the *DMPK* gene. For this purpose, a prenatal test was carried out, revealing a normal allele and another with about 195 CTG repeats (Figure 1. III.4), not being possible to perform a Southern blot analysis to quantify the expanded allele due to insufficient prenatal DNA sample. In view of these results, and with the necessity of ruling out a possible germline mosaicism before thinking about a phenomenon of contraction in the number of copies, Southern blot was conducted after birth. The quantification of the expanded allele revealed 333 CTGs (as her father, without having expanded the CTG repeat size) (Figure 2B), which made us discard both the possibility of a mosaicism and a contraction phenomenon in the number of CTG repeats.

## Discussion

DM1 is transmitted in an autosomal-dominant inheritance pattern, so the risk of each child of an affected patient inheriting the mutation is 50%. Being the expanded CTG repeat in the abnormal range at the DM1 locus one of the most unstable sequences in the human genome, with germline length change mutation rates often greater than 95%, it tends to increase in size in successive generations. That is why children may inherit repeat lengths considerably longer than those present in the transmitting parent. This phenomenon is known as genetic anticipation, in which disease severity increases and/or age of onset decreases from one generation to the next [8, 9]. CTG repeats are also highly unstable in the soma, and mutations appear to accumulate through multiple small-length changes [7]. Thus, it seems rational to assume that the expansion-biased, age-dependent and tissue-specific nature of somatic instability contributes towards both the tissue specificity and progressive nature of the symptoms [9]. Clinically, anticipation has been a strikingly consistent phenomenon in a large number of DM1 families. In this study, we report the case of a family with several intergenerational expansions of the CTG repeat, in which once one of the members was diagnosed with DM1, close medical and family vigilance resulted in earlier diagnosis of the disease in the offspring, allowing a better management of the patient and an appropriate genetic counselling for family planning.

It is known that congenital DM1 occurs mostly with maternal transmissions of the mutation (specially from expanded alleles of >300 CTG repeats), generally accompanied by a large intergenerational increase of the CTG repeat size. This family case, with a congenital form observed through maternal transmission of the CTG repeat expansion, supports this fact and also the postulation of some maternal factor in the pathogenic mechanism of congenital DM1.

However, the CTG repeat size does not always increase in successive generations of DM1 families, and characteristics of these cases have not been systematically studied. With an estimated population frequency of about 4.2–6.4%, reported in a single study over 20 years ago, a decrease in the CTG repeat size during transmission from parents to child has also been described, mainly during paternal transmissions (10%). We report here a case with paternal transmission, in whose affected daughter a contraction phenomenon was initially suspected. This first suspicion, which arose after prenatal testing as the TP-PCR showed a peak of around 195 CTG repeats, was then discarded by Southern blot after birth. This case emphasizes the importance of Southern blot in the diagnostic confirmation procedure for DM1; since, although TP-PCR cheaper and more accessible for most laboratories, it determines if the length of the allele is in the normal or pathological range, but it does not allow to accurately quantify the number of repeats of more than 150 to 200 CTGs [10]. The low frequency of occurrence of the contraction phenomenon (never reported in our laboratory after more than 550 genetic studies of DM1) moves us to highlight the importance of discarding false identifications due to both germinal mosaicisms and expansions over 150 CTG repeats that escape from most TP-PCR kits.

In DM1, repeat expansion length is predictive of clinical severity and age of onset. Going back to our case, even if the expanded paternal allele did not experience a contraction phenomenon in the offspring, it is quite curious the fact that father and daughter have exactly the same number of CTG repeats.

In conclusion, this case highlights both the limitation of the screening techniques (fluorescent PCR or TP-PCR) in the diagnostic procedure of DM1, bringing to light the essential role of confirmatory methods (Southern blot) and the important role that plays the sex of the affected parent in the determination of the CTG repeat size in the offspring. Even if the estimated progenitor allele length is the major modifier of disease severity in DM1 accounting for 64% of the variation in age at onset in a simple log-linear relationship [9], there may be an additional destabilizing maternal factor that allows the CTG repeats to further expand, increasing the probability of congenital DM1 in the offspring, and a paternal factor that restricts the expansion and prevents the occurrence of congenital DM1. Undoubtedly, more studies are needed to fully clarify the mutational dynamics associated with expanded sequence repeats loci, including the identification of additional participating genetic modifiers. This knowledge together with a greater understanding of the genotype–phenotype relationship in DM1 and related unstable microsatellite disorders will facilitate the genetic stratification, prognostic assessment and clinical management of these patients and their families.
